# Dementia Caregivers' Trajectories of Well-Being Before and During the COVID-19 Pandemic

**DOI:** 10.1093/geroni/igab046.1207

**Published:** 2021-12-17

**Authors:** Lauren Mitchell, Elizabeth Albers, Robyn Birkeland, Colleen Peterson, Anna Drake, Joseph Gaugler

**Affiliations:** 1 Emmanuel College, Boston, Massachusetts, United States; 2 University of Minnesota, Minneapolis, Minnesota, United States; 3 University of Minnesota, University of Minnesota, Minnesota, United States

## Abstract

Residents of long-term care settings and their family caregivers have been severely impacted by the COVID-19 pandemic. The present mixed-methods study examines trajectories of well-being pre- and post-pandemic onset for caregivers of persons with dementia living in residential long-term care. Participants were taking part in, or had recently completed, an ongoing intervention trial to support families transitioning a relative into long-term care. Beginning in summer 2020, we started assessing caregivers’ COVID-19-related experiences and added three surveys spanning 4-months beyond the 12-month parent study period to capture caregivers' adjustment throughout the pandemic. Using latent growth curve models, we estimated caregivers' (N = 104) trajectories of depressive symptoms, burden, and self-efficacy before and during the pandemic. We also tested whether the counseling intervention had protective effects for participants in the treatment group, and examined moderators including long-term care facility size, care recipient's dementia and health status, and quality of staff interactions.

